# A Monoblock Light-Scattering Milk Fat Percentage and Somatic Cell Count Sensor for Use in Milking Systems

**DOI:** 10.3390/s23208618

**Published:** 2023-10-21

**Authors:** Alexey V. Shkirin, Maxim E. Astashev, Dmitry N. Ignatenko, Nikolai V. Suyazov, Sergey N. Chirikov, Vladimir V. Kirsanov, Dmitriy Y. Pavkin, Yakov P. Lobachevsky, Sergey V. Gudkov

**Affiliations:** 1 Prokhorov General Physics Institute of the Russian Academy of Sciences, Vavilova st. 38, Moscow 119991, Russia; astashev@yandex.ru (M.E.A.); dmitriyek13104@yandex.ru (D.N.I.); nvsnvs@list.ru (N.V.S.); s_makariy@rambler.ru (S.V.G.); 2 Laser Physics Department, National Research Nuclear University MEPhI, Kashirskoe sh. 31, Moscow 115409, Russia; snchirikov@mephi.ru; 3 Federal State Budgetary Scientific Institution “Federal Scientific Agroengineering Center VIM”, 1st Institutsky Proezd 5, Moscow 109428, Russia; kirvv2014@mail.ru (V.V.K.); dimqaqa@mail.ru (D.Y.P.); lobachevsky@yandex.ru (Y.P.L.); 4 Institute of Biology and Biomedicine, Lobachevsky State University of Nizhny Novgorod, Gagarin av. 23, Nizhny Novgorod 603105, Russia

**Keywords:** optical sensors, laser light scattering, milk quality control, milking systems

## Abstract

A monoblock light-scattering sensor, which is capable of measuring the fat content of milk and indicating the excess by which the somatic cell count (SCC) is over the permissible level, has been developed for installation in dairy systems. In order for the sensor to perform measurements when the milking machine is working in the “milk plug” mode, a flow-through unit is designed in the form of a pipe with a lateral cylindrical branch, in which milk accumulates so as to eliminate large bubbles and achieve continuity of the milk flow. The operation of the sensor is based on the registration of the angular intensity distribution of light scattered in the transparent cylindrical segment of the tube branch. A semiconductor laser with a wavelength of 650 nm is used as a light source for determining scattering in milk. The angular distribution of the scattered light intensity (scattering indicatrix) is recorded using an axial photodiode array. The fat content is determined by the average slope of the measured scattering indicatrix in the range of scattering angles 72–162°. The SCC level is estimated from the relative deviation of the forward scatter intensity normalized to the backscatter intensity with respect to uninfected milk. The sensor has been tested on a Yolochka-type milking machine.

## 1. Introduction

Automated control of milk composition is an important factor in the efficiency of dairy production, including dairy farms, where sensing technologies are increasingly being used, gradually replacing expensive and time-consuming classical invasive chemical methods. Express analysis of the percentage of milk components (fat, proteins, lactose, amino acids, progesterone, etc.), as well as microbiological impurities and somatic cells, provides the necessary information for assessing milk quality parameters, which enables farmers to establish a balanced feed for cows and diagnose their clinical condition [1,2,3]. The primary indicator of milk quality is the content of somatic cells, which is characterized by the somatic cell count (SCC), that is, the number of cells in a milliliter of milk. According to the results of veterinary studies, an excess of SCC above 10^5^ per mL is regarded as a sign that the cow is infected with mastitis [4,5]. At the same time, the market value of milk depends chiefly on the content of fat and protein. Thus, real-time monitoring of the composition of milk is essential for a prompt response to deviations in the parameters characterizing the physiological state of animals. This causes the demand for low-cost, in-line milk composition sensors that can be compactly integrated into dairy equipment.

Control devices intended to be built into milking systems must satisfy the requirement that there should not be a significant pressure drop inside the milk hose [6,7]. Therefore, optical methods for analyzing milk composition seem to be the most suitable base for designing milk composition sensors intended for dairy equipment on farms, due to their high sensitivity and speed, as well as the possibility of non-invasive, non-contact diagnostics [8,9,10,11,12,13,14,15]. The operational quantitative assessment of milk composition is carried out, as a rule, using optical spectroscopy instruments [16]. Particularly, Fourier transform infrared (FTIR) spectroscopy is used in high-precision systems, which, however, are very expensive and generally bulky. Apart from FTIR systems, less expensive and more compact spectroscopic near-infrared (NIR) milk composition analyzers are increasingly being used [17,18,19]. As well as spectroscopic milk analyzers, light-scattering milk composition sensors are very promising, since they can be made compact, fast and cheap, while providing sufficient accuracy in measuring the percentage of fat and protein. Although there are several studies suggesting different schemes for applying light scattering to the determination of the component percentages in milk [20,21,22,23,24], commercial offers for light-scattering milk composition sensors are currently lacking. Due to the dispersive properties of milk, light scattering sensors intended for integration into milking systems have to work in conditions of multiple scattering. Single-scattering-based milk analyzers require milk dilution [25] and, thus, are not suitable for in-line monitoring of milk parameters. Forward- and side-scatter measurements are used to determine the SCC in milk via flow cytometric analysis [26,27]; however, flow cytometry is not non-invasive and requires milk sample treatment. Multiple light scattering in the NIR range can also be informative, in particular for the monitoring of milk fermentation [28]. For the analysis of particle size and concentration in dense media, including food stuffs, instruments are offered that employ methods such as photon density wave (PDW) spectroscopy [29,30,31] and static multiple light scattering (SMLS) [32,33,34], but they are configured to work with closed cuvettes and a small set of scattering angles. We propose a somewhat new approach to the light scattering diagnostics of turbid media such as milk, which consists of tracking changes in the shape of the angular distribution of the scattered light intensity (scattering indicatrix), being measured in a wide range of scattering angles from forward- to backscatter, due to changes in the content of milk components such as fat and large-scale impurities (in milk, this role is played by somatic cells). Moreover, as experiments show, the ratio of contributions to the scattering intensity from fat particles and somatic cells varies at different scattering angles, which allows them to be separated to determine the content of these components. This regularity was reported in our previous work [35].

This work is a continuation of our previous study [35], where we proposed a prototype of an in-line sensor for measuring the fat percentage and indicating the concentration level of somatic cells in milk flowing through a pipeline. We have significantly modified the original design of the sensor, the novelty of which includes several aspects. Firstly, a flow-through unit with a lateral pipe branch was designed to operate the sensor in the milk plug mode. Secondly, we have developed a monoblock sensor layout, in which the controller and the optical unit are placed on the same board. Thirdly, a new mathematical algorithm is proposed for determining the fat content from the scattering indicatrix. It is based on the calculation of the average slope of the indicatrix in the range of scattering angles 72–162° using the least-squares method, which takes less controller resources than the polynomial approximation of the dependence of the intensity difference between forward- and backscatter on fat content, implemented in [35]. In this work, we carried out a theoretical simulation showing that the addition of large particles (an order of magnitude larger than milk fat droplets) has the greatest effect on the forward scattering intensity and does not practically perturb the intensity of side- and backscattering, from which the fat percentage of milk is derived. This fact substantiates the use of the relative deviation of the forward scatter intensity for detecting the excess content of somatic cells in milk.

Our device has some advantages, such as the fact that the optical measuring system is combined with the controller in one block, which sends the values of the measured parameters of milk composition directly to the output RS 232 serial port. An open format for transferring the milk parameters implies the possibility of integrating the device into milking systems, regardless of their type and the manufacturer. It is worth emphasizing the importance of the sensor being able to operate in the milk plug mode, that is, when an air–milk mixture separated by air gaps moves in the milk hose. In order for the light-scattering technique to provide sufficient measurement accuracy, it is necessary to make the milk flow continuous. It should be noted that earlier in the development of the NIR spectroscopic sensor [19], a design feature was implemented for the sampling chamber, which enables us to minimize the effect of milk flow pulsations and turbulence on the measurement accuracy. This sampling chamber is shaped as a recessed cavity adjoining the main milk flow conduit from a downward direction, so that it is filled with a constantly renewed sample of the flowing milk. In our prototype of the light-scattering sensor, this problem is solved in a rather new way by adding a lateral cylindrical pipe branch adjacent to the main milk pipe in the flow-through unit of the sensor and having a common longitudinal slot with the main pipe. In the pipe branch, the movement of the milk plug slows down, which leads to its continuous filling with milk. To enable angle-resolved light scattering measurements, a transparent cylindrical segment is embedded into the pipe branch concentrically, along with which a semicircular array of eight photodetectors is installed.

Whereas the fat content of milk is measured by our light-scattering sensor with good accuracy (the absolute error of fat percentage is in the order of tenths), the measurement error of the SCC is high. Therefore, the proposed sensor is not intended to replace conventional methods for detecting somatic cells in milk, such as milk conductivity measurement, microscopy of a milk sample and inspection of individual parts of the udder, and can only be used as an indicator of deviation from the mean SCC level corresponding to “uninfected” case.

## 2. Sensor Design

### 2.1. Device Layout

**Figure 1:**
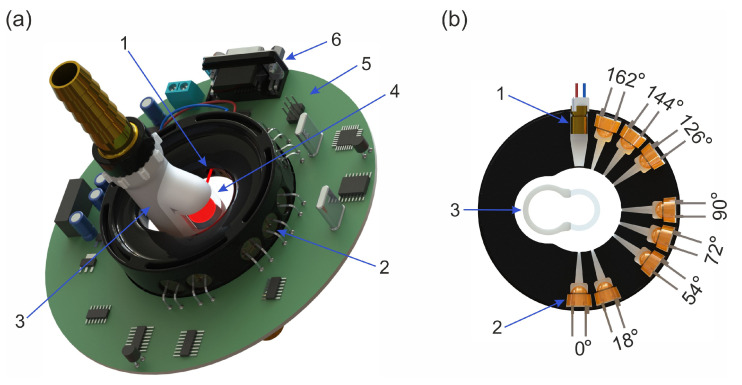
Device layout. (**a**) Components: (1) laser diode emitting at a wavelength of 650 nm, (2) axially arranged photodiodes, (3) flow-through unit, (4) transparent cylindrical segment, (5) controller board, (6) RS 232 serial port. (**b**) Optical system arrangement: (1) laser, (2) photodiodes, (3) flow-through unit.

We have implemented a prototype of our milk composition sensor, which is based on angle-resolved light scattering measurements and has a monoblock layout. The layout of the sensor is shown in Figure 1. It includes an optical unit, a microcontroller and a flow-through unit.

The optical unit consists of a laser diode with a wavelength of 650 nm and a power of 3 mW and an axial photodiode array. The laser beam is directed along the diameter of a cylindrical segment made of fused quartz with an outer diameter of 15 mm and a wall thickness of 2 mm. The scattering of the laser radiation by milk filling the quartz segment results in the appearance of a certain angular distribution of light intensity around it. To record the scattering indicatrix, a semicircular array of eight silicon photodiodes arranged in a ring holder with an angle step of 18° is used. The ring holder has an outer diameter of 68 mm. The part of the holder where the photodiodes are inserted has a semicylindrical inner surface with a diameter of 30 mm. The photodiodes have a 3 × 3 mm^2^ photo-receiving area molded into a plastic housing with a hemispherical lens. Photodiodes are located axially with respect to the lateral cylindrical pipe channel in three groups for receiving the scattered light in the angular regions of forward scattering (0°, 18°), side scattering (54°, 72°, 90°) and backscattering (126°, 144°, 162°). In front of each photodiode, the scattered light field is narrowed by a rectangular diaphragm with a slot width of 1 mm, which is cut out from the inner surface of the annular photodiode holder.

The photodiodes FD263-01 from Chipdip (Moscow, Russia) are connected in the photoconductive mode to the transimpedance amplifier OP482 from Texas Instruments (Dallas, TX, USA) with a 1MOhm feedback resistor. The ATMEGA328P microcontroller is used with the AD7705 analog-to-digital converter to sample the photodiode signals. To take into account the level of the dark current, the microcontroller sets the laser module to a square-wave operation mode. From the photodiode data, the controller calculates the milk composition parameters using the firmware and sends them to the output RS232 port.

**Figure 2:**
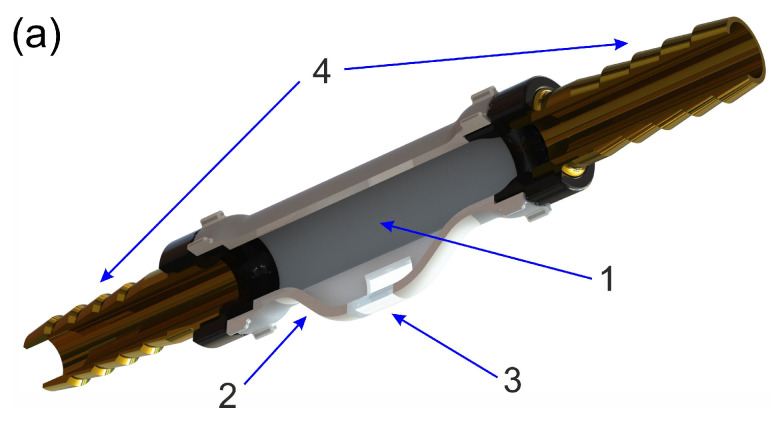

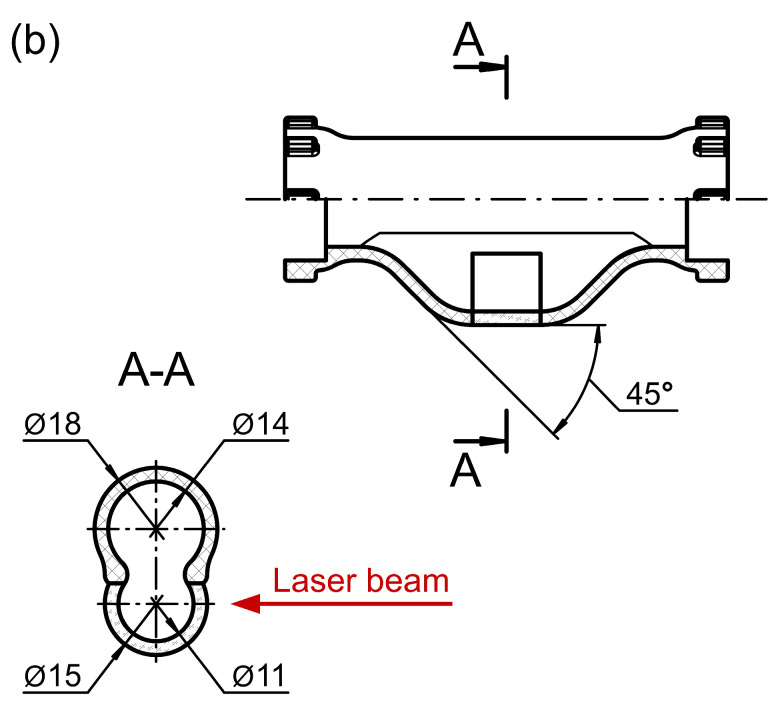
Design of the flow-through unit. (**a**) 3D view: (1) main milk pipe, (2) lateral cylindrical pipe branch, (3) transparent cylindrical segment of fused quartz, (4) herringbone fittings. (**b**) Side view and cross section of the pipe chamber.

The design of the flow-through unit is illustrated in detail in Figure 2.

In order to achieve a continuous milk flow in the measuring chamber, a lateral pipe channel is added to the main pipe at a branching angle of 30°, so that it has a common slot with the main pipe, as is shown in Figure 2b. In the lateral channel, the milk flow slows down and becomes continuous. To make the measuring chamber transparent to laser radiation, a cylindrical quartz segment is inserted into the lateral channel, which serves as an optical cell for observing angle-resolved light scattering using a semicircular photodetector array, mounted concentrically with the quartz segment. The milk samples continuously refill the channel when the milking machine is in operation. The measuring chamber of the flow-through unit was built from polyamide plastic by means of FDM 3D printing technology. The fittings terminating the ends of the main channel are removable and can be chosen according to the diameter of the milk hose used.

**Figure 3:**
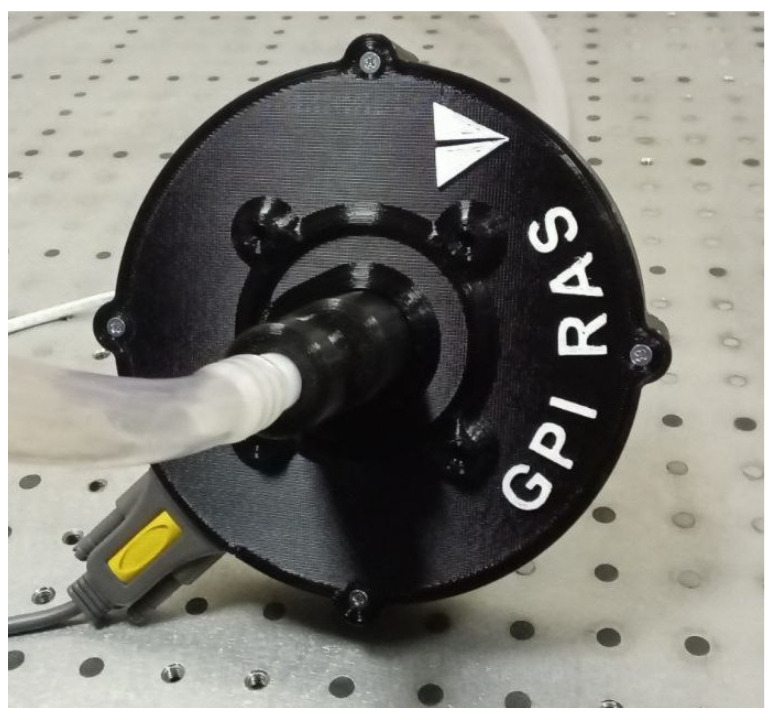
Monoblock light-scattering milk fat percentage and SCC sensor assembled in the housing.

Assembled in a housing, the sensor has an overall dimension of 14 cm; its overall view is shown in Figure 3.

### 2.2. Principle of Operation

The photodiode array receives light scattered by milk, which is illuminated by a laser diode oscillating in the square-wave mode with a pulse width of 5 s. The photocurrent readings are taken at a frequency of 1 s^−1^ and subjected to median filtering. For each of the eight photodiodes located at the scattering angles (0°, 18°, 54°, 72°, 90°, 126°, 144°, 162°), the difference between the median of the photocurrent when the laser is emitting and the median of the dark current is recorded as the scattering intensity value with an update rate corresponding to the square-wave switching of the laser. According to the logarithmic values of the scattering intensity measured at the scattering angles (72°, 90°, 126°, 144°, 162°), the average slope of the scattering indicatrix is determined using the least-squares method [36]. Then, by substituting the obtained slope value into the established characteristic function related to the fat content of milk, the fat percentage is determined. In addition, the relative deviation of the difference in logarithmic intensity between forward- (0°) and backscatter (specifically, 144°) angles from the calibrated difference value for uninfected milk is calculated. The value of this deviation is compared with the mean value corresponding to the admissible SCC of 10^5^ per mL to indicate that the SCC level is exceeded. The controller makes the necessary calculations in the cycle mode and constantly updates output values for the fat percentage and intensity difference deviation.

## 3. Materials and Milking Equipment

### 3.1. Milk Samples and Somatic Cells

In order to calibrate the sensor in terms of the milk fat percentage, we have conducted experimental tests with a series of cow milk samples with different fat percentages: 0.05%, 1.5%, 3.0%, 4.5% and 6%. This set of fat percentages was obtained through successively mixing two primary milk samples commercially produced, using the technology of UHT pasteurization and homogenization (skimmed milk with a nominal fat content of 0.05%, and high-fat milk with a nominal fat content of 6%) in equal proportions. The protein content of the primary milk samples was reported as 3.0 g per 100 g of product.

To determine the sensitivity of the sensor to the presence of large particles in milk such as somatic cells, we examined the scattering indicatrix recorded by the sensor when MCF-7 human breast carcinoma cells (ATCC no. HTB-22) with an average size of ~20 µm were added to milk samples. These cells can be considered a suitable proxy for the somatic cells that appear in the milk of mammals (cows in particular) when infected with mastitis. Using a pipette, the cells were suspended in the studied milk samples to achieve the resulting concentrations: 5 × 10^4^, 10^5^, 5 × 10^5^ and 10^6^ cm^−3^.

### 3.2. Milking Equipment

**Figure 4:**
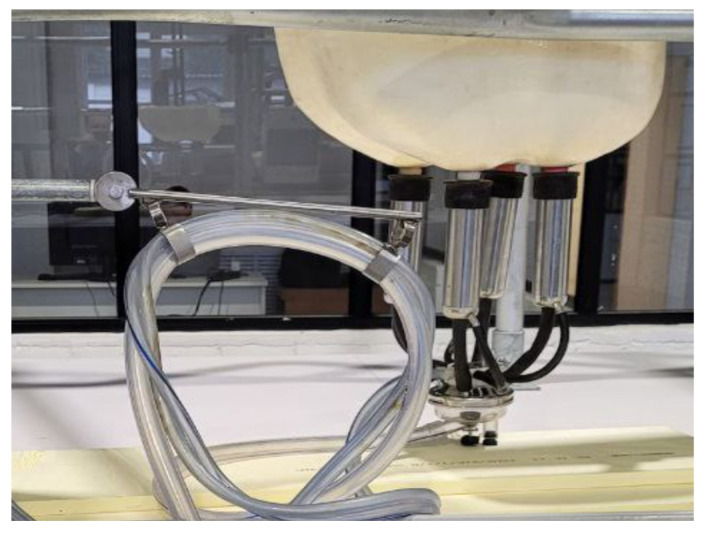
Experimental stand based on a modified Yolochka-type milking system.

The experiments with the prototype sensor in milking mode were carried out using an experimental stand based on a modified Yolochka milking system, which is shown in Figure 4. Milking systems of the Yolochka type are a classic basis for modern dairy farming, proven for decades to be robust and efficient thanks to the benefits of individual milking site equipment and excellent throughput [37,38]. The experimental stand includes a mock-up of a cow’s udder, a typical milking machine and a special connection for installing a flow-through sensor.

The vacuum-producing system ensures a stable vacuum level of 47 ± 1 kPa in the milking mode and 50 kPa in the flushing mode. It consists of polymer pipes and a rotary vane vacuum pump with oil lubrication. Maintaining the vacuum pressure in the vacuum and milk lines of the milking machine is carried out using a vacuum regulator.

The maximum flow of the milk–air mixture in the milk hose is 100 mL/s, which corresponds to the actual milking regime with maximum parameters. The flow of the milk–air mixture flows in the milk hose with incomplete and uneven filling, as in production conditions. The milking cups of the experimental stand are connected to an anatomical model of an udder filled with milk. The actual filling of the milk hose with milk during milking is never complete; in most cases, the filling is from one-third to two-thirds of the full volume. Therefore, integrated measuring modules for milk quality analysis should be developed while taking into account the circumstance that the flow of milk in the milking machine is not uniform, and that there is an alternation of milk and air plugs.

## 4. Results

### 4.1. Fat Percentage Measurement

**Figure 5:**
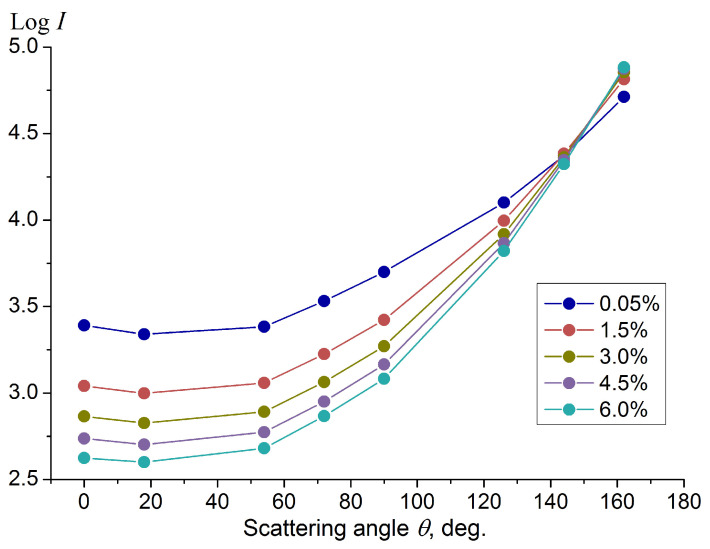
Scattering indicatrices (scattering intensity *I* vs. scattering angle *θ*) measured for samples of UHT milk with a fat percentage of 0.05%, 1.5%, 3.0%, 4.5% and 6% at zero flow velocity in the cylindrical quartz segment under normal conditions. The wavelength of light is 650 nm. The experimental error of the intensity logarithm is no more than 0.02.

The angular distribution of the scattered light intensity (the scattering indicatrix) was measured for a series of UHT cow milk samples with a fat percentage of 0.05%, 1.5%, 3.0%, 4.5% and 6% using a laser diode emitting at a wavelength of 650 nm together with an axial array of eight photodiodes located at the scattering angles (0°, 18°, 54°, 72°, 90°, 126°, 144°, 162°) according to the scheme described in Section 2. At the first stage, aimed at calibrating the sensor, light scattering measurements were carried out in a static case, that is, for non-flowing milk filling a cylindrical quartz segment with an outer diameter of 15 mm and an inner diameter of 11 mm (depicted in Figure 2) under normal conditions (pressure 1 atm and temperature 20 °C). The scattering indicatrices for milk with different fat contents, obtained by averaging over three independently prepared samples of each fat content level, are shown on a logarithmic scale in Figure 5. The root-mean-square error of the scattered intensity logarithm does not exceed 0.02.

The angular behavior of the scattering intensity in Figure 5 showing an increase from the forward scattering angles to the backscattering ones is typical for a multiple scattering medium. This characteristic shape of the scattering indicatrix is confirmed via theoretical modeling for multiple scattering media [39,40]. This is due to the large optical depth of the milk sample, exceeding the value of 10 in the case of filling a pipe with an inner diameter of 11 mm, which assumes a multiple scattering regime [41]. The local rise in the intensity values recorded at the strict forward scattering angle (0°) is associated with the diffuse transmission of the laser beam.

**Figure 6:**
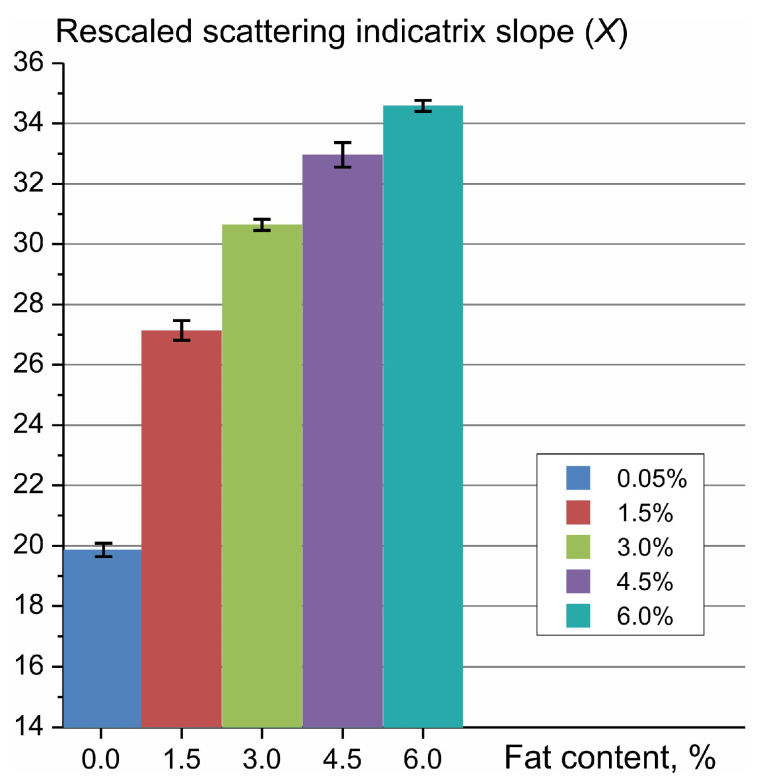
Rescaled slope of the logarithmic scattering indicatrix on the angular interval (72–162°) *X*, calculated using the least-squares method for UHT milk with different fat contents in the range of 0–6% using the data shown in Figure 5 after multiplying by a factor of 1548.

Similar to the results of the light scattering measurements obtained earlier for milk in cylindrical geometry [35], the scattering intensity values change monotonically with an increase in the fat percentage of the milk, leading to a different slope of the scattering indicatrix in the angular range from backscatter to side scatter. Thus, the slope of the dependence of the scattering intensity logarithm on the scattering angle can be taken as a parameter that is sensitive to the fat percentage. This slope can be determined in a simple way by determining the difference in the scattering intensity logarithm between the side scattering angles (72° or 90°) and backscattering angles (144° or 162°), as was performed earlier [35]. Here, we propose a more accurate way to determine the slope by calculating the linear regression coefficients, which are determined by the least-squares method [36] for the logarithmic values of the scattering intensity $Log{(I}_{scat})$ measured at the scattering angles 72°, 90°, 126°, 144° and 162°. To streamline calculations within the controller, the angle variable has been rescaled so that the slope values become substantially larger than 1 to avoid using floating point operations. The slope values X obtained for the studied milk samples in the fat content range of 0–6% are shown in Figure 6; the data are presented as mean and standard deviation.

As can be concluded from Figure 6, the scattering indicatrix slope increases by about 1.7 times with an increase in fat content from 0 to 6%. Meanwhile, the logarithm of the scattering intensity at an angle of 162° increases only by 1.04 times with a similar increase in fat content. This fact, together with the property of the scattering indicatrix that its shift as a whole along the vertical axis (which can occur due to a number of factors, such as an instability in the laser power) does not affect the calculation of the slope and, accordingly, the fat content thus measured, makes the method of measuring milk fat content via the indicatrix slope more attractive than via backscattering intensity alone.

**Figure 7:**
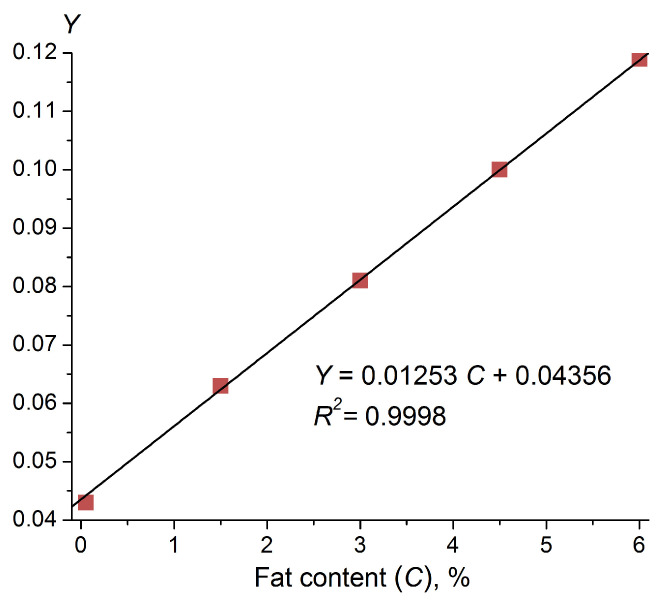
Linearization of the characteristic curve for the fat content in UHT milk. Y is the slope function given by Formula (1).

The dependence of the scattering indicatrix slope on the fat content is non-linear. However, it can be linearized by a simple functional substitution:

(1) $$Y=\frac{1}{a-X^{\prime }}$$

where X is the slope of the logarithmic scattering indicatrix; a is an experimentally adjusted parameter (for UHT milk a = 43); and Y is the characteristic function of the sensor for measuring the fat content of milk, capable of linearization with sufficient accuracy. Applying linear regression in accordance with the least-squares algorithm, we obtained a formula for the linear dependence of Y on the fat content of UHT milk:

(2) $$Y=0.01253\;C+0.04356,\;R^{2}=0.9998,$$

where C is the fat percentage of the milk, and $R^{2}$ is the coefficient of determination. The corresponding characteristic plot of the sensor’s response to milk fat content is displayed in Figure 7.

As in the case of the unbranched flow-through unit of the sensor described in [35], the static calibration defined by the characteristic plots (Figure 6 and Figure 7) remains valid for a laminar milk flow at a flow rate of up to 100 mL/s. By testing the sensor with a Yolochka milking machine connected to cow’s udder mock-up filled with 2.5% UHT milk, it was found that the absolute error in measuring the fat percentage, experimentally determined for the milk plug mode, is about ±0.5%. This error is associated with a certain turbulence of the milk flow in the measuring chamber.

**Figure 8:**
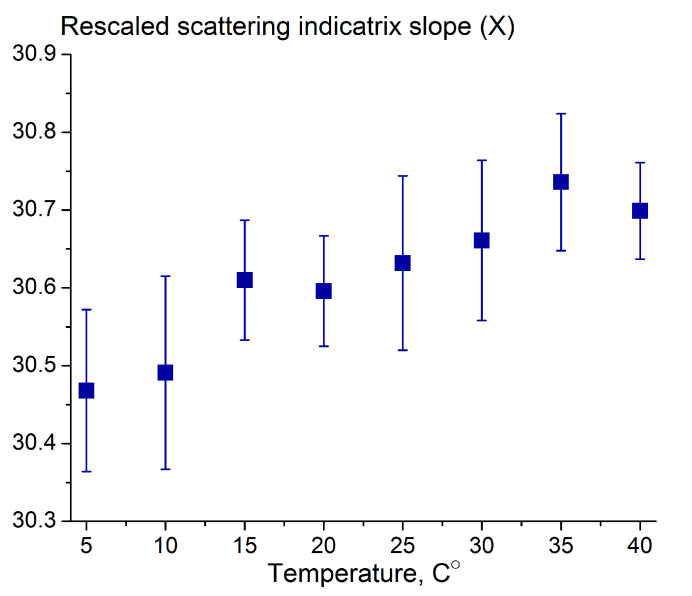
Variation of the scattering indicatrix slope with temperature in the range of 5–40 °C for UHT milk with 3% fat. Averaging over three independently prepared milk samples was performed.

In practice, the temperature of the milk in the hose can vary significantly depending on external conditions; therefore, we measured the variation of the scattering indicatrix slope with temperatures in the range of 5–40 °C, taking as an example UHT milk samples with 3% fat (Figure 8). Sample thermostating was carried out using a Biosan TS-100C thermoshaker (Riga, Latvia).

It turned out from Figure 8 that the slope increases very slowly with the temperature and results in an absolute error in fat content of no more than 0.1% based on the characteristic plot for the sensor (Figure 7).

### 4.2. SCC Estimation

**Figure 9:**
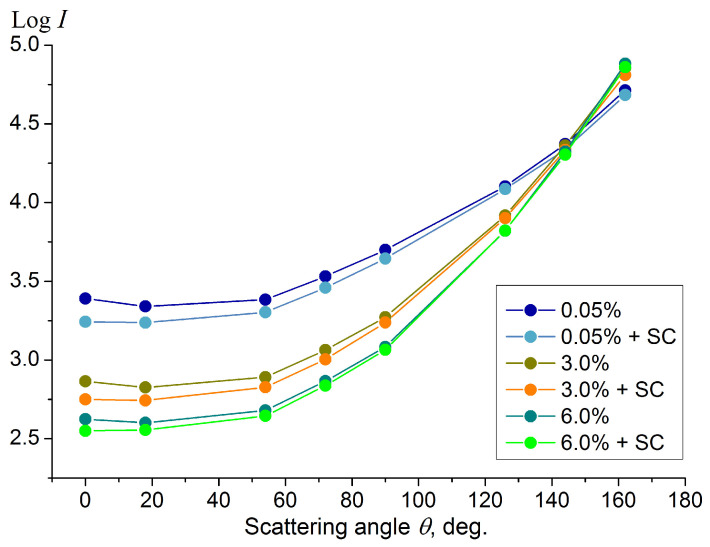
Scattering indicatrices (scattering intensity I vs. scattering angle θ) measured for UHT milk samples with a fat percentage of 0.05%, 3.0% and 6.0% with and without the addition of somatic cell (SC) proxies at a concentration level of 10^6^ cm^−3^. Milk flow velocity in the pipe is zero. Laser wavelength is 650 nm.

In order to study the response of the light scattering indicatrix to the presence of somatic cells and the possibility of assessing the SCC in milk using the developed sensor, we carried out comparative measurements of the light scattering indicatrix in UHT milk samples of different fat contents with and without the addition of somatic cell (SC) proxies at a resulting concentration of 10^6^ cm^−3^ (Figure 9).

As can be seen from the graphs in Figure 9, the presence of large-scale impurity particles such as SC proxies in milk has a specific effect on the shape of the scattering indicatrix, leading to a significant deviation in the light intensity only at forward scattering angles. This is an important fact for the proposed method of fat content measurement in milk, since the presence of cells will not significantly distort the measured fat percentage values derived from the slope of the scattering indicatrix in side- and backscattering because the average rate of change in the indicatrix at these angles remains virtually unchanged. Slope calculations obtained from the data in Figure 9 on the angular interval of 72–162° show that the relative deviation of the slope caused by an SCC level of 10^6^ per mL is 0.042 and 0.024 for UHT milk with 3% and 6% fat, respectively. In accordance with the characteristic plot (Figure 7), this deviation leads to an absolute error in measuring the fat content of no more than 0.15%. The SCC-induced slope deviation can be reduced if a smaller angular interval is used to calculate the slope. For example, the relative deviation of the slope calculated within the interval of 90–162° would be 0.006 and 0.003 for UHT milk of 3% and 6% fat, respectively. As the fat content of the milk increases, the effect of adding the cells on the scattering indicatrix begins to gradually decrease, apparently due to their screening by the growing amount of fat particles.

Similar to [35], we characterize the deviation of the scattering indicatrix due to large impurity particles by the intensity drop from backscattering to forward scattering, namely, the difference between the intensity logarithms. Such a difference parameter is independent of laser power fluctuations. A scattering angle of 144° seems to be the most suitable as a reference point in the backscattering, near which the indicatrices for different fat content intersect. Thus, we have introduced a scatterometric parameter as follows:

(3) $$S\left(\theta \right)=LogI\left(144{}^{\circ}\right)-LogI\left(\theta_{i}\right),\;\theta_{i}=\left\{0{}^{\circ},18{}^{\circ}\right\}.$$

**Figure 10:**
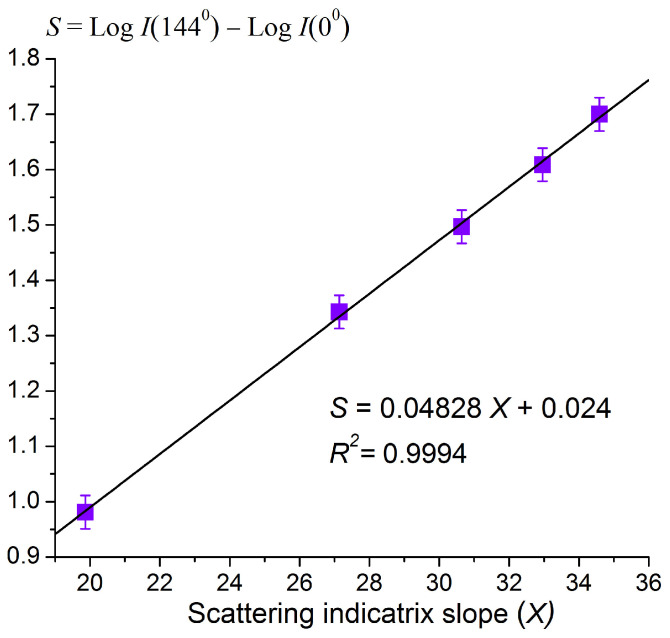
Dependence of the scatterometric parameter *S* at a scattering angle of 0° on the scattering indicatrix slope determined in the angular interval (72–162°) for UHT milk.

In Figure 10, the dependence of the scatterometric parameter at a scattering angle of 0° on the scattering indicatrix slope X at angles of 72–162° for UHT milk is plotted. X, in turn, is uniquely related to the fat percentage of the milk (Figure 6).

The data in Figure 9 are easily approximated via a linear function:

(4) $$S=0.04828X+0.024,\;R^{2}=0.9994,$$

where X is the scattering indicatrix slope determined in the angular interval (72–162°), and R² is the coefficient of determination. The function (4) can be used as a reference level to calculate the scatterometric parameter deviation caused by the appearance of somatic cells in milk.

As a parameter sensitive to the presence of large-scale impurity particles in milk, which is directly measured by the sensor, we have assigned the index of large particles LPI, determined as the relative deviation of the scatterometric parameter from the value corresponding to uninfected milk:

(5) $$LPI=\frac{S-S_{pure}}{S_{pure}}.$$

**Figure 11:**
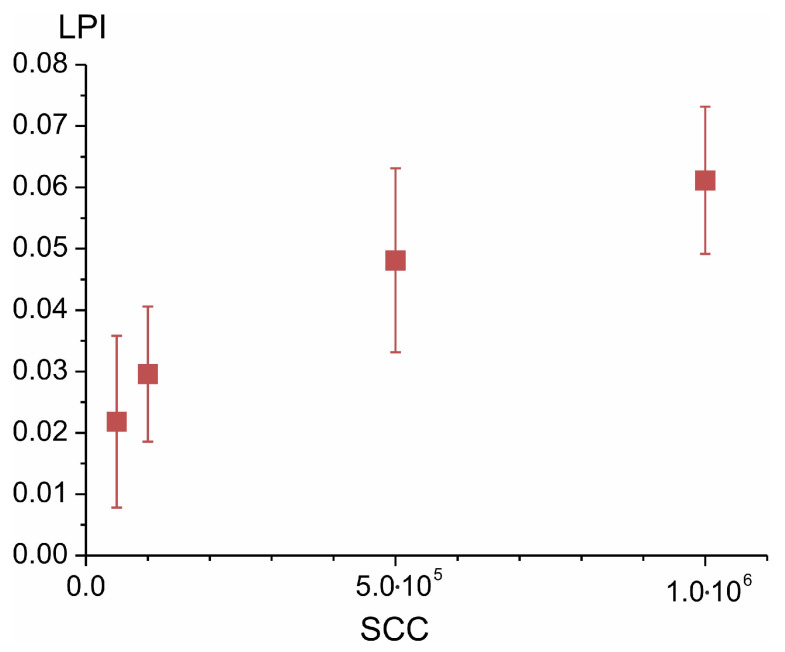
Dependence of SCC on the LPI value measured by the sensor, which is obtained by adding SC proxies with concentrations of 5 × 10^4^, 10^5^, 5 × 10^5^, 10^6^ cm^−3^ to samples of uninfected UHT milk with a fat content of 3.0%.

We measured the LPI for various concentrations of SC proxies added to UHT milk with a fat content of 3.0% in the range of 5 × 10^4^–10^6^ cm^−3^. The LPI measurements were averaged over three independently prepared milk samples with suspended SC proxies at each concentration. Thereby, we have established the relationship between the SCC and LPI (Figure 11).

However, due to the large random error in the LPI values, the SCC estimate obtained from LPI would be inaccurate. From the plot in Figure 10, it follows that in this way, it is possible to register only a significant excess of milk SCC over the allowable level of 100,000 per mL. This means that in relation to the SCC, the sensor can only work as an indicator of the SCC deviation from the normal case. Alternatively, averaging over a long interval of time during in-line measurements can improve the reliability of the average LPI measurements and, thus, better estimate the SCC level.

### 4.3. Test Measurements with the Sensor for Raw Milk: Uninfected *versus* Mastitis Case

**Figure 12:**
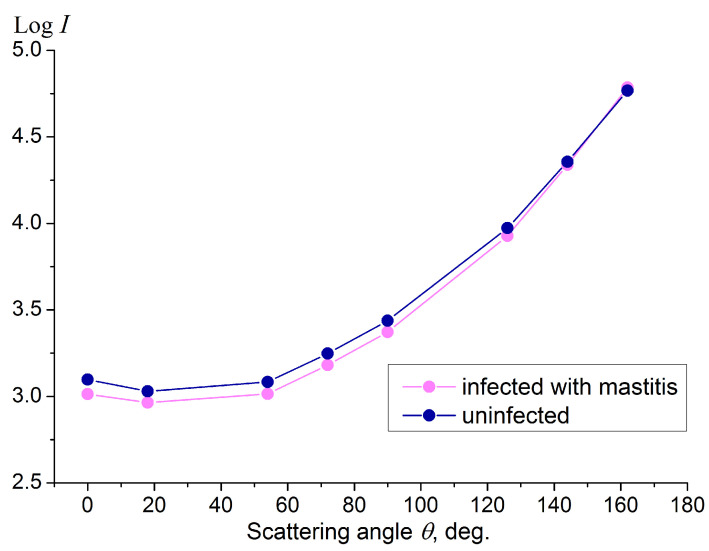
Scattering indicatrices (scattering intensity I vs. scattering angle θ) measured for raw milk samples obtained from healthy (uninfected) cows and from a cow infected with mastitis. Fat content was 3.8% and 4.3% with SCC < 200,000 per mL and ~500,000–700,000 per mL, respectively, for the uninfected and infected case. Milk flow velocity in the pipe is zero. Laser wavelength is 650 nm.

To determine the ability of the sensor to distinguish mastitis milk from uninfected milk, we conducted tests on raw milk samples taken from a dairy farm. The quality parameters of the milk samples collected from cows were assessed using a reference ultrasonic device for milk quality analysis, Laktan 1-4M, manufactured by Sibagropribor (Moscow, Russia), which allows you to measure the fat percentage in a milk sample with an accuracy of hundredths. The level of SCC in milk was determined using a Kenotest measuring kit produced by CID Lines NV (Ieper, Belgium). We selected two raw milk samples to test the light-scattering sensor: the first (obtained from healthy cows) had a fat content of 4.3% and SCC < 200,000 per mL; the second (obtained from a cow infected with mastitis) had a fat content of 3.8% and SCC ~ 500,000–700,000 per mL. The scattering indicatrix plots measured for these milk samples in a stationary case (zero flow rate) are shown in Figure 12.

Firstly, a comparison of Figure 5 and Figure 12 shows that the indicatrix of uninfected raw milk is higher than the indicatrix for UHT milk of the corresponding fat content. This is explained by the fact that the size of fat droplets in raw milk is larger than in UHT milk. According to data from [42], the average droplet size in raw milk is 3.00 ± 0.91 μm, and in UHT milk it is 1.35 ± 0.38 μm. Consequently, the number of fat drops in UHT milk is greater than in raw milk with the same fat content; therefore, UHT milk is optically more turbid than raw milk of the same fat percentage. This means that to correctly measure the fat content of raw milk, it is necessary to recalibrate the sensor using a procedure similar to that which we applied to the UHT milk, that is, using a series of raw milk samples of a given fat content prepared through the sequential dilution of high-fat raw milk. In addition, we found that raw milk samples acquired from different dairy farms show good reproducibility of the scattering indicatrices measured by the sensor at the same fat content; therefore, sensor calibration, once performed on raw milk, will be valid regardless of the specific animal. Secondly, in the mastitis case, the scattering indicatrix deviates downward in relation to the indicatrix of the uninfected raw milk. The relative deviation of the scatterometric parameter (Equation (5)) at a scattering angle of 0° is about 0.05. Thus, the trend in light scattering by raw milk observed in a real case of mastitis infection (Figure 12) correlates with the results obtained for UHT milk with artificial additives of somatic cells (Figure 9).

## 5. Discussion

A prototype sensor based on light scattering in cylindrical geometry for in-line measurement of fat content and assessment of the SCC level in milk was implemented in a monoblock layout. The sensor has a fairly simple design and consists of cheap optoelectronic components such as a semiconductor laser diode and silicon photodiodes. The housing parts and tubes can easily be made from plastic. All parameters of the proposed prototype, including the elements of the controller, are specified to facilitate replication of our results.

As an original technical solution to the problem of milk flow continuity arising in the milk plug mode of milking machine operation, it was proposed to place the measuring chamber in a lateral branch pipe that communicates with the main pipe. A cylindrical quartz segment was embedded into the pipe branch to input the laser beam and receive the light scattered by the milk. It should be noted that this design of the measuring chamber is well adapted for the rinsing of the milking system without stagnation of liquid in standard mode, due to the branching angle of 45°. Additionally, we proposed a new technique for determining the fat content based on the calculation of the slope of the scattering indicatrix in the angular range of the side- and backscatter. The experiments revealed that the light scattering indicatrix responds both to changes in the concentration of fat drops, which determines the fat percentage of the milk, and to the addition of large-scale impurities. At the same time, the presence of large particles in the milk (whose size exceeds the average size of fat droplets by an order of magnitude) mainly changes the scattering intensity of forward scattering without practically affecting side- and backscattering, while the fat percentage is derived from the behavior of the scattering indicatrix only in the region of side and backscatter. Thus, fat percentage measurements turned out to be resistant to the presence of large particles in milk. Note that not only somatic cells, but also large clots of fat and air bubbles can act as large particles in raw milk. Therefore, for a correct in-line assessment of the SCC via light scattering, it is first necessary to determine the average background value for the index of large particles in uninfected milk (when there is no infection with mastitis), relative to which the change introduced by somatic cells should be counted. Replacing the photodiode, located at a scattering angle of 0°, with a camera that provides greater angular resolution at small forward scattering angles can increase the sensitivity of the sensor to the presence of somatic cells in milk, but this will complicate the device and increase its cost.

We have developed a method for calibrating the sensor by preparing successive dilutions of high-fat milk mixed with skimmed milk. A comparison of measurement results for UHT milk and raw milk ascertained that although each of these cases requires a different calibration of the sensor for fat content, the presence of somatic cells in milk leads to a similar effect on the light scattering pattern, which was tested on UHT milk with artificial additives of somatic cell proxies as well as on raw milk taken from a cow infected with mastitis.

It is also essential to note that the weak influence of the milk’s temperature on the light scattering characteristics makes temperature correction unnecessary, thereby maintaining the simplicity of the device.

The theoretical simulation of light scattering in a spherical volume of milk (see Appendix A) showed that there is a monotonous variation of the scattering indicatrix at the forward- and side scattering angles with an increase in the fat content of the milk, and the addition of large particles results in a downward deviation of the forward scattering intensity. The same kind of scattering intensity deviation caused by the addition of somatic cells to milk was observed experimentally using the sensor. In general, the simulation confirmed the possibility of separating the contributions to the scattering indicatrix from fat particles and large impurities over different angular scattering regions, thus allowing for the simultaneous determination of the fat content and SCC level.

Tests of the sensor connected to a milking system of the Yolochka type operating in the milk plug mode showed that the absolute error of in-line determination of the fat percentage in milk does not exceed tenths of a percent.

Since there are few proposals for in-line milk quality sensors on the dairy equipment market, we believe that sensors based on light scattering have a high competitive potential and deserve increased research interest. The primary direction for improving the measuring accuracy of the proposed sensor should be aimed at further optimization of the measuring chamber geometry (volume, branching angle) in order to minimize the turbulence and bubble formation in the milk flow inside the chamber that occurs in the milk plug regime. The functionality of the sensor can be expanded by installing a UV LED emitting at a wavelength of 280 nm, which effectively excites casein fluorescence. The casein content can be directly quantified from the fluorescence intensity using semiconductor photodetectors. Lastly, the developed prototype sensor can be considered a low-cost in-line analyzer, at least for a preliminary assessment of milk quality; thus, the methodology for calibrating and verifying the sensor using reference methods should be an important aspect of future research.

## 6. Conclusions

The implemented model of a light-scattering sensor can serve as a prototype for a relatively small-sized and inexpensive device for in-line monitoring of the fat percentage and SCC level in milk. Such a sensor, which outputs the fat percentage and SCC level directly to a serial RS 232 port, is capable of integrating with the milking system controller in a conventional way and, thus, can be used in milking parlors and automated milking systems (AMS) with milking robots.

## Figures and Tables

**Figure A1:**
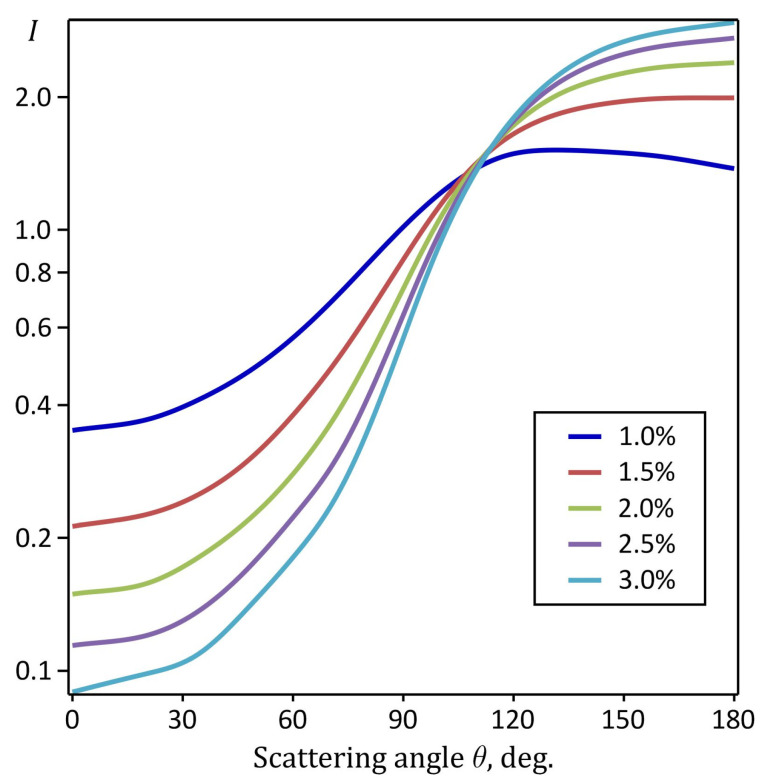
Scattering indicatrices (scattering intensity I vs. scattering angle θ) simulated for milk of different fat percentages in a spherical volume with radius $R_{V}$ = 0.55 cm.

**Figure A2:**
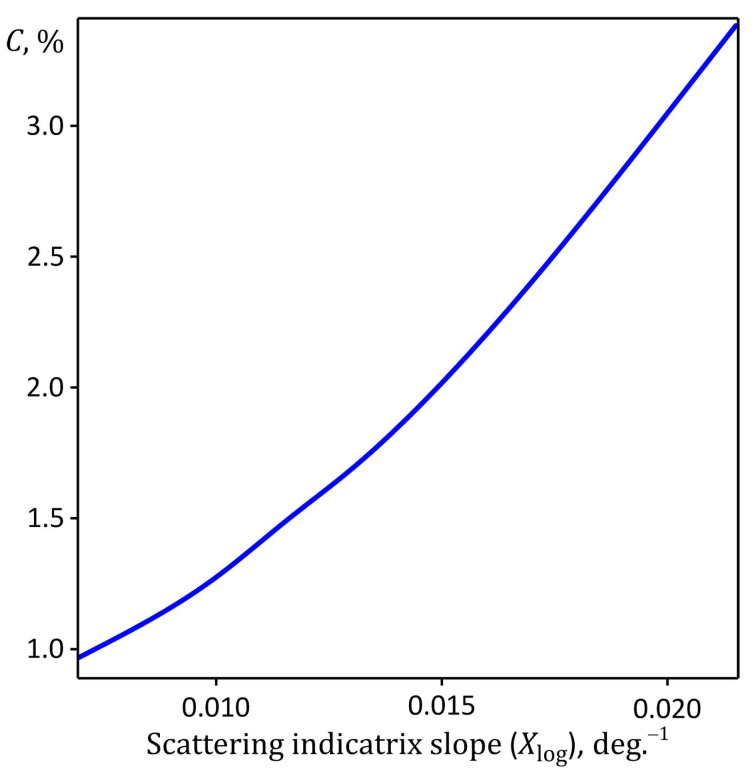
Dependence of the fat percentage of milk C on the average slope $X_{log}$ of the decimal logarithm of the scattering indicatrix. Numerical simulation was performed for a spherical scattering volume with radius $R_{V}$ = 0.55 cm.In this angular range, a twofold increase in the slope $X_{log}$ corresponds to approximately the same twofold increase in fat content.

**Figure A3:**
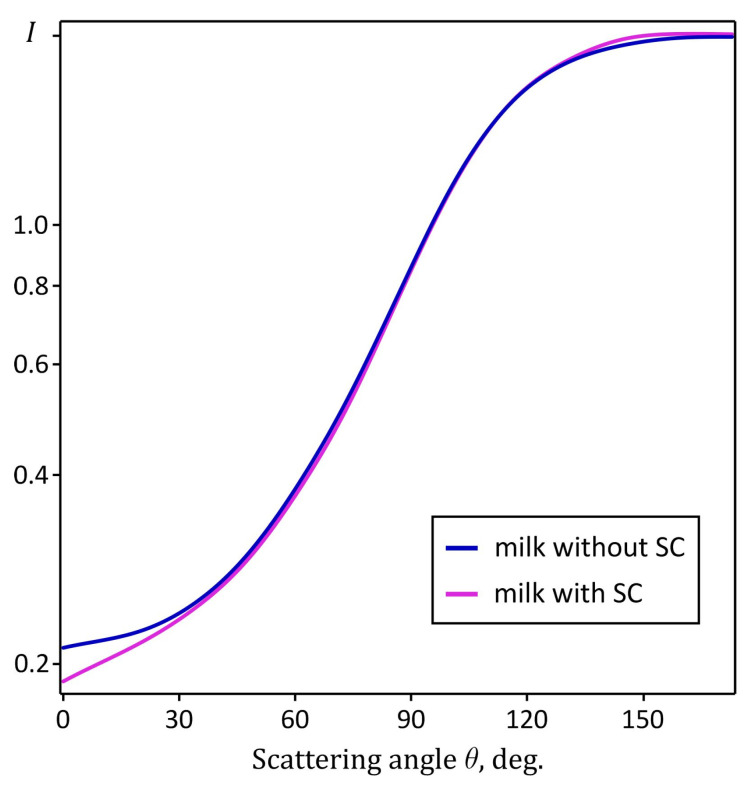
Simulated dependence of the intensity of light scattered in a spherical volume of milk on the scattering angle: with the addition of somatic cells with a concentration of $N_{SC}$ = 3 × 10^6^ cm^−3^ (milk with SCs) and in the absence of such cells (milk without SCs). Numerical modeling was performed for the volume radius $R_{V}$ = 0.55 cm and a fat content of 1.5%.

## Data Availability

Not applicable.

## Appendix A. Theoretical Simulation of Light Scattering in Milk

In order to theoretically confirm the light-scattering effects underlying the proposed method for determining fat content and detecting somatic cells, numerical modeling of the scattering of optical radiation with a wavelength of 650 nm in a two-component turbid medium consisting of fat droplets and large impurities was performed. The size of the impure particles was taken to be an order of magnitude larger than that of fat droplets. The emulsion of milk fat globules was modeled by spheres with a relative refractive index m = 1.1 and an average radius of 1.3 µm, which is close to the average value typical for milk [42]. As a model of somatic cells, spheres with m = 1.03, having an average radius of 13 µm, were used. The individual scattering matrices for the particles of the simulated medium were calculated using the T-matrix algorithm developed in [43].

For concentrations of fat particles corresponding to milk with a fat content ≥ 1%, the multiple-scattering regime is in effect. We simulated this regime numerically using the Monte Carlo method. In this case, the calculation algorithm described in [44] was taken as a basis. There, a volume containing scattering particles, which is limited by a spherical surface of radius $R_{V}$, is considered. The use of spherical symmetry makes it possible to significantly reduce the number of test photons and, consequently, the time of numerical simulation. It should be noted that the light-scattering sensor proposed in our work has a scattering volume geometry that is different from a spherical one. Therefore, we do not carry out a quantitative comparison of the results of numerical simulation with the results of the experiment, but we believe that a qualitative comparison of the obtained dependences can explain the properties of the scattering indicatrix in milk, which are used in the operating algorithm of the sensor. We have modified the algorithm [44] for use in the case of a two-component medium, when there are two types of scatterers, fat globules and somatic cells, which differ significantly in size, specifically, by an order of magnitude. In addition, the simulation results showed that at a fat content of the order of 1% or more and a radius $R_{V}$ ≥ 0.5 cm, the optical radiation is highly depolarized already near the entrance to the scattering volume (the registered photons are subjected to at least 10^2^ scattering events on average). This allows us to consider the radiation as unpolarized in the entire volume of the medium. Such an approximation makes it possible to reduce the time of the numerical simulation by about four times and, as a comparison of the calculation results showed, does not introduce noticeable changes in the calculated scattering intensity.

The results of the numerical simulation of multiple scattering in a medium that models milk filling a spherical volume with a radius of $R_{V}$ = 0.55 cm are shown in Figure A1, Figure A2 and Figure A3. Here, the scattering intensity is normalized as follows:

(A1) $$\frac{\pi }{360{}^{\circ}}\int_{0}^{180}I\left(\theta \right)sin\theta d\theta =1,$$

where θ is the scattering angle in degrees (I is the relative fraction of power scattered into a solid angle of one steradian).

Figure A1 displays the angular dependences of the intensity of the light scattered in a spherical volume on the scattering angle, modeled for various fat percentages of milk.

The family of the simulated scattering indicatrices plotted on a logarithmic scale in Figure A1 shows that with a change in fat content, the slope of these indicatrices undergoes the most noticeable change in the angular region close to 75°. Therefore, as an indicator of fat content, the value of their average slope $X_{log}$ in an interval from an angle near 75° to a certain backscattering angle can be taken:

(A2) $$X_{log}=\frac{{d\left[LogI\left(\theta \right)\right]}_{lin}}{d\theta },$$

where ${\left[LogI\left(\theta \right)\right]}_{lin}$ is the result of indicatrix linearization on the interval [$\theta_{1}$, $\theta_{2}$]. For example, we took $\theta_{1}$ = 75° and $\theta_{2}$= 115°. As shown in Figure A2, the fat content is unequivocally determined by the value $X_{log}$.

The change in the scattering indicatrix of milk with the addition of scatterers that model somatic cells is illustrated in Figure A3.

This addition leads to a clearly noticeable decrease in the scattering intensity at forward scattering angles in the region θ < 30°. At the same time, the value of the parameter $X_{log}$, associated with the fat content of milk, undergoes a relative increase of only 0.025, that is, it remains practically unchanged and can lead to an error in determining the fat percentage only in the third decimal place.

Thus, the numerical simulation confirms the constructiveness of the approach to the light scattering analysis of milk composition, based on the selection of individual angular regions for detecting different dispersed components of milk.
